# Epidemiology of hip & knee replacement across Pakistan: Multicenter cross-sectional study

**DOI:** 10.12669/pjms.39.6.7006

**Published:** 2023

**Authors:** Syed Imran Bukhari, Asad Rehman Allana, Kazim Rahim Najjad, Syed Shahid Noor, Amin Chinoy

**Affiliations:** 1 Syed Imran Bukhari, FCPS. Department of Trauma & Orthopedics, Lady Reading Hospital, Peshawar, Pakistan; 2 Asad Rehman Allana, BDS, DRS. Department of Community Health Sciences, Aga Khan University Hospital, Karachi, Pakistan; 3 Kazim Rahim Najjad, FCPS. Department of Trauma & Orthopedics, Liaqat National Hospital, Karachi, Pakistan; 4 Syed Shahid Noor, FRCS. Department of Trauma & Orthopedics, Liaqat National Hospital, Karachi, Pakistan; 5 Amin Chinoy, FRCS. Department of Trauma & Orthopedics, Indus Hospital, Karachi, Pakistan

**Keywords:** Joint registry, Knee replacement, Hip replacement

## Abstract

**Objective:**

To know about the predictive prevalence of hip and knee arthroplasty across Pakistan.

**Methods:**

It is retrospective cross-sectional study with data collection from Pakistan National Joint Registry (PNJR) of number of hip and knee arthroplasty cases in seven years (2014-2021). Fourteen high volume centers across Pakistan who consented for data publication were included. Stata version 16 was used for data analysis. Mean & standard deviation was reported for quantitative variable & frequency and proportion were reported for qualitative variables.

**Results:**

Our results showed a total of 9572 people had total knee replacement in last seven years from 2014-2021 with the rate of 9.57/100,000 population. Mean age of the patient was 61.7±8.95 with 69.5% patients being female and 30.5% being male. Our results showed a total of 2265 people had total hip replacement in last seven years from 2014-2021with the rate of 2.26/100,000 population. Mean age of the patient was 50.7±15.4 with 62% patients being male and 38% being female.

**Conclusion:**

This is the first epidemiological study in Pakistan on the rates of hip and knee arthroplasty cases in Pakistan based on registry data, showing that more knee arthroplasty cases are being performed as compared to hip arthroplasty.

## INTRODUCTION

Knee and hip osteoarthritis are prevalent conditions that necessitate frequent follow-up, medical therapy, and potentially costly procedures such as joint replacement surgery. There is little information available about the prevalence of these pathologic diseases in the general population. Currently, researchers attempting to determine the prevalence of knee or hip osteoarthritis must rely on medical histories, radiographs, or symptom descriptions across the hospitals using hospital management information system.^1^ Total hip and knee replacements are universally accepted as safe and effective treatments for degenerative joint disease.^2^

The most frequent underlying disease for both knee replacement & hip replacement is osteoarthritis. Inflammatory arthritis, fracture, dysplasia, bone tumor and other disorders can also lead to knee replacement & hip replacement. Though the outcomes of knee & hip replacement fluctuate due to variances in joint structure and underlying medical conditions, most patients receive considerable long-term improvement with both surgeries. The number of patients living with hip & knee replacements has increased significantly over the last two decades, and after several decades of running joint arthroplasty services, the number of patients with multiple revised joints has also increased.^3^,^4^

Age, weight, gender, previous joint trauma, joint dysplasia, and unaccustomed sports activity are all risk factors for its development. Age appears to be the most common factor. This is because chondrocyte function declines with age, as does the ability to synthesize adequate proteoglycan aggregates. These proteoglycan aggregates are less sensitive to cytokines and mechanical stresses, predisposing cartilage to degeneration.^5^,^6^ Another major factor is obesity. Obesity causes the repetitive application of high axial loading stresses on the surface of a joint, resulting in articular cartilage degradation. Obesity also causes articular cartilage to develop irregularly and impairs its healing.^7^,^8^

According to statistics from Brazilian patients undergoing THR or TKR, osteoarthritis was the major reason for both surgeries, and hypertension was the most common comorbidity among patients.^9^ With higher rates of advanced arthritis diagnosis and treatment, and growing demand for improved mobility and quality of life, annual procedure volumes are expected to rise significantly in the coming decades, making joint replacements the most common elective surgical procedure.^7^ Over a 10-year period (2003–2013), Australia reported a 105 % rise in primary TKR utilization and a 73 % increase in THR surgery.^10^,^11^ Kurtz et al. Anticipated 673 % growth in TKR and 174 % growth in THR in the United States from 2005 to 2030.^12^

Hip and knee joint replacement surgeries are among the most common health-care procedures performed worldwide. Saeed et al concluded that total hip arthroplasty begets tremendous benefits.^13^ We sought to estimate the current prevalence and historical trends in the prevalence of total hip and total knee replacement in Pakistan using the Pakistan national joint registry (PNJR; https://www.arthroplasty.org.pk/about-pnjr/) which was launched in February 2014 while accounting for the recent secular increase in procedure volumes and their success using the Knee society score and the Harris hip score. This will be first of its kind study on this topic in Pakistan.

### Research question:

1. What is prevalence of TKA and THA according to Pakistan National Joint Registry (PNJR)?
2. What were the indications for TKA and THA?
3. Is there a difference in the distribution of procedures across Pakistan?
4. What type of implants were used in hip & knee arthroplasty?

## METHODS

It is a retrospective cross-sectional study of 14 high volume centers across Pakistan with information of patients who had undergone TKA & THA in last seven years obtained through PNJR. Those hospitals who registered with PNJR and had signed memorandum of understanding on patient confidentiality and data protection and who consented to data publication were part of this study. The study duration was seven years (2014-2021). Ethical review was taken via IRD_IRB_2014_03_004, Oct 19, 2016. Non-probability convenience sampling was used.

### Inclusion criteria:

1. Both gender
2. Patient of any age
3. Patient with all the comorbid conditions
4. Patients undergoing TKA and THA

### Exclusion criteria:

1. Patients with incomplete/missing records (e.g., age, BMI, Harris hip scores/Knee society scores and comorbidities.

### Data collection and analysis

Data was collected through PNJR. Data was analyzed using Stata version 16. Mean & standard deviation was reported for quantitative variable & frequency and proportion were reported for qualitative variables. The rate of primary THR and TKR per 100,000 was computed by dividing the number of procedures in the registry by the relevant catchment population from the Pakistan national joint registry for a given age, gender, and year.

## RESULTS

Our results showed a total of 9572 people (Table-I) had total knee replacement in last seven years from 2014-2021 with the rate of 9.57/100,000 population. Mean age of the patient was 61.7±8.95 with 69.5% patients being female and 30.5% being male. Around 72.6% patients were from Sindh, 22.6% patients were from Punjab and 4.7% patients were from KPK. About 69.2% patients had comorbidities like hypertension while 25.2% patients had diabetes. Post-operative complications like hematoma were reported in 0.06% of patients while DVT & pulmonary embolism was reported in 0.03% of patients.

**Table-I:** Shows frequency of patients with total knee replacement, sociodemographic features and the factors associated with total knee replacement.

S.NO	Variables	Frequency n (%)	Percentage
	Total	9,572	80.9%
1	Age* (Median IR))	61.7±8.95	
2	Weight* (Mean±SD)	80.7±19.6	
3	BMI* (Mean±SD)	30.5±7.48	
4	***Gender***		
	Male	2,893	30.5%
	Female	6,577	69.5%
5	***State***		
	KPK	454	4.7%
	Punjab	2,166	22.6%
	Sindh	6,952	72.6%
6	***Comorbidities***		
	Diabetes	2,412	25.2%
	Asthma	237	2.5%
	Hypertension	6,622	69.2%
	Ischemic Heart Disease	439	4.6%
	Hepatitis	91	0.9%
	CKD	50	0.5%
7	Previous Knee Surgery	75	0.5%
8	***Complications***		
	Deep Vein Thrombosis	3	0.03%
	Dislocation	2	0.02%
	Fracture	2	0.02%
	Hematoma	6	0.06%
	Infection	2	0.02%
	Fracture	2	0.02%
	Dislocation	2	0.02%
9	***Knee society score***		
	Poor	8,935	93.5%
	Fair	463	4.8%
	Good	145	1.5%
	Excellent	17	0.2%
10	***Functional score***		
	Poor	9,078	94.9%
	Fair	302	3.2%
	Good	158	1.7%
	Excellent	27	0.3%
11	***Type of implant***		
	Zimmer	2,823	29.5%
	Biomet	1,200	12.5%
	DePuy	2,560	26.7%
	MicroPort	24	0.25%
	Implant cast	18	0.30%
	Surgival	36	0.48%
	U-2	157	1.6%
	Johnson & Johnson	242	2.52%
	Smith & nephew	349	3.6%
12	Thromboprophylaxis		
	Heparin	2,707	28.3%
	Warfarin	6	0.06%
	Direct thrombin indicators	71	0.73%
	Aspirin	146	1.5%
13	Ambulator		
	Community ambulator	6,845	78.6%
	Home ambulator	1,716	19.7%
	Non-ambulator	143	1.64%
14	Ambulatory support		
	With support	3,812	46.6%
	Without support	4,364	53.4%

* Data reported for only those patients whose data was available. Missing data excluded.

Before undergoing surgery 93.5% patients had poor knee society score, 4.8% patients had fair scores and 1.5% patients had good knee society scores while 0.2% had excellent knee society scores. Poor functional score was reported in 94.9% of patients. In 42% of patients, Zimmer Biomet implant was used while in 29.2% patients Depuy implant was used. The percentage of patients who were community ambulator was 78.6% while 19.7% of patients were home ambulator.

Our results showed a total of 2265 people (Table-II) had total hip replacement in last seven years from 2014-2021 with the rate of 2.26/100,000 population. Mean age of the patient was 50.7±15.4 with 62% patients being male and 38% being female. Punjab accounted for 34.8% of patients, 37.8% of patients were from Sindh and 27.3% of patients were from KPK. Hypertension was reported in 31.9% of patients while 9.80% of patients had diabetes. Femur fracture was reported in 0.22% of patients while 0.09% of patients had dislocation in follow-up. The mean Harris hip score was 18.37±0.51. Corail by Depuy was the predominant implant used. The percentage of patients who were community ambulator was 64.2% while 28.4% patients were home ambulator.

**Table-II:** Shows frequency of patients with total hip replacement, sociodemographic features and the factors associated with total hip replacement.

S.NO	Variables	Frequency n (%)	Percentage
	Total	2,265	19.13%
1	Age* (Mean±SD)	50.7±15.4	-
2	Weight* (Mean±SD)	74.5±17.4	
3	BMI* (Mean±SD)	26.3± 6.9	
4	***Gender***		
	Male	1,377	62.0%
	Female	843	37.9%
5	***State***		
	KPK	618	27.3%
	Punjab	789	34.8%
	Sindh	858	37.8%
6	***Comorbidities***		
	Diabetes	222	9.80%
	Asthma	32	1.41%
	Hypertension	724	31.9%
	Ischemic Heart Disease	73	3.22%
	Chronic kidney disease	10	0.44%
	Hepatitis	27	1.20%
	Rheumatoid	1	0.33%
8	***Type of Implant***		
	Actinia	70	3.10%
	Corail	500	7.50%
	Self-locking	121	5.34%
	Zimmer	1	0.04%
	United	97	4.30%
	Versys	41	1.81%
	S2	64	2.82%
	Paragon	6	0.30%
	Muller	21	0.93%
9	Harris hip score	18.37± 0.51	
10	***Complications***		
	Infection	1	0.04%
	Dislocation	2	0.09%
	Fracture	5	0.22%
	Wound	1	0.04%
11	***Ambulator***		
	Community ambulator	1,111	64.2%
	Home ambulator	492	28.4%
	Non-ambulator	129	7.5%
12	***Ambulatory support***		
	With support	895	62.5%
	Without support	537	37.5%
13	***Thromboprophylaxis***		
	Heparin	726	84.7%
	Warfarin	0	0
	Direct thrombin indicators	30	3.5%
	Aspirin	93	10.9%
	Panta	3	0.35%

* Data reported for only those patients whose data was available. Missing data excluded.

## DISCUSSION

Our study is first of its kind conducted in Pakistan reporting total number of cases of total knee and hip replacement, socio-demographic features and other relevant factors associated with replacements in last seven years from 2014-2021 using Pakistan national joint registry data base. Our results showed the rate of 9.57 and 2.26 of total knee and hip replacement, this corresponds to 11837 patients in last seven years. Our study showed the mean age of patients with total knee replacement to be around 61.7±8.95 and of hip replacement to be around 50.7±15.4. A study conducted by Jones et al. showed similar results for total knee arthroplasty as 86% of the patients were between 55 and 79 years of age.^14^

Our results showed that mean weight of patients with total knee replacement was 80.7±19.6 and mean weight of patients with total hip replacement was 74.5±17.4. A study conducted by Robert et al. showed 75% of the THA recipients and 88% of the TKA recipients were overweight.^15^ Our result showed that 30.5% of males and 69.5% females were TKA recipients while 62% males and 38% females were THA recipients. A study conducted by Kurtz et al. showed similar results for TKA recipients as the rates of knee arthroplasties were 67% higher for female compared to male but showed opposite results for THA recipients as the rates were 36% higher for female compared to males.^12^

Our study showed that 69.2% TKA recipients had comorbidities like hypertension, 25.2% patients had diabetes, 4.4% patients had ischemic hearts disease while 31.9% THA recipients had hypertension, 3.22% patients had ischemic heart disease & 9.80% patients had diabetes. A study conducted by Podmore et al. showed that at least one comorbidity was found in 63.6% of hip arthroplasty patients and 71.3 % of knee arthroplasty patients. The most common comorbidity was high blood pressure (48.4 % in hip arthroplasty patients and 57.1 % in knee arthroplasty patients), followed by heart disease (17.1% and 18.5%), lung disease (13.9% and 15.6%), and diabetes (9.5 % and 13.6 %, respectively).^16^

Our results showed that in TKA recipients the major complications included deep vein thrombosis (0.03%), hematoma (0.06%) and infection (0.02%) while in THA recipients the major complications included infection (0.04%), dislocation (0.09%), fracture (0.22%). A study conducted by Jain NB et al. showed that the odds of having post-operative complications like DVT, infection increases in the patients who are diabetic, hypertensive and obese.^17^

Another study conducted by Courtney et al. showed that chronic obstructive pulmonary disease (adjusted OR 4.16, 95 percent confidence interval: 1.86-9.32), congestive heart failure (adjusted OR 9.71, 95 percent confidence interval: 4.55-20.71), coronary artery disease (adjusted OR 2.80, 95 percent confidence interval: 1.38-5.69), and cirrhosis (adjusted OR 8.43, 95 percent confidence interval: 1.63-43.59) were all risk factors for post-operative complications.^18^ Our results showed that 84.7% of patients with TKR received heparin thromboprophylaxis and 10.9% patients received aspirin thromboprophylaxis. While 28.3% of patients with THR received heparin thromboprophylaxis and 1.5% patients received aspirin thromboprophylaxis.

A study conducted by Schousboe JT et al showed that venous thromboembolism (VTE) was recorded in >35% of patients undergoing total joint arthroplasty in the absence of prophylaxis, despite the fact that the majority were asymptomatic. The ideal technique for preventing VTE after total joint arthroplasty is of debate, with some doctors strongly favoring the use of newer anticoagulants,^19^ while recent research papers in high-volume centers favoring aspirin in low-risk patients and low-molecular weight Heparin in high-risk patients.

Our results showed that among TKR recipients, 42% patients had Zimmer biomet implant, and 29.2% had DePuy implant. A study conducted by Kahlenberg et al. showed that in 5.1% patients Zimmer biomet was used while in 18.8% NexGen was used with patients in the NexGen group were the most likely to be satisfied (odds ratio (OR) 1.63; p = 0.006).^20^ Our study showed that among THR recipients Corail by Depuy was the most commonly used implant (7.50%). A study conducted by Fevang et al. showed that the best results were obtained with the cemented prosthesis (Depuy & zimmer).^21^

The strength of our study is that the data was taken from PNJR database over a period of seven years from 2014 to 2021which included patients from all over Pakistan with different socio-economic status, different hospitals, different surgeons and all the factors affecting the TKR and THR replacement procedures giving detailed description of all the factors affecting the results of both procedures, so the results can be generalized to whole population.

### Limitation

Entering data in PNJR is voluntary so all the cases of TKR and THR are not present in the registry affecting our final estimate of all TKR and THR patients in Pakistan in last seven years. Another limitation of this study is that data of some patients was incomplete as factors such as age, height, BMI for patients were missing resulting in overall very low mean creating discrepancy in our final estimates. Finally, due to varying hospital policies, some did not consent to data publication.

## CONCLUSION

Our results showed that in last seven years 9572 patients were TKR recipients and 2265 patients were THR recipients. The major factors leading to significant increase in TKR and THR include high prevalence of arthritis, growing demand for greater mobility & quality of life and effectiveness of joint replacement surgeries in recent decades. On the other hand, the high prevalence of joint replacement necessitates a competent management of this population’s long-term health-care needs, as well as a reduction in the burden of complications and reoperations. Targeted research and the implementation of effective, evidence-based policies can fulfill these demands.

### Authors Contribution:

**SIB:** Conceived and designed the study.

**ARA:** Did the statistical analysis.

**KRN, AC:** Did the data collection.

**SSN:** Did the review and is responsible for integrity of research.
